# LNCipedia 5: towards a reference set of human long non-coding RNAs

**DOI:** 10.1093/nar/gky1031

**Published:** 2018-10-29

**Authors:** Pieter-Jan Volders, Jasper Anckaert, Kenneth Verheggen, Justine Nuytens, Lennart Martens, Pieter Mestdagh, Jo Vandesompele

**Affiliations:** 1 Cancer Research Institute Ghent (CRIG), 9000 Ghent, Belgium; 2 Center for Medical Genetics (CMGG), Ghent University, 9000 Ghent, Belgium; 3 VIB-UGent Center for Medical Biotechnology, 9000 Ghent, Belgium; 4 Department of Biomolecular Medicine, Faculty of Medicine and Health Sciences, Ghent University, 9000 Ghent, Belgium

## Abstract

While long non-coding RNA (lncRNA) research in the past has primarily focused on the discovery of novel genes, today it has shifted towards functional annotation of this large class of genes. With thousands of lncRNA studies published every year, the current challenge lies in keeping track of which lncRNAs are functionally described. This is further complicated by the fact that lncRNA nomenclature is not straightforward and lncRNA annotation is scattered across different resources with their own quality metrics and definition of a lncRNA. To overcome this issue, large scale curation and annotation is needed. Here, we present the fifth release of the human lncRNA database LNCipedia (https://lncipedia.org). The most notable improvements include manual literature curation of 2482 lncRNA articles and the use of official gene symbols when available. In addition, an improved filtering pipeline results in a higher quality reference lncRNA gene set.

## INTRODUCTION

The human genome is pervasively transcribed, producing vast amounts of RNA transcripts, of which the majority does not encode protein (1). Long non-coding RNAs (lncRNAs) are typically defined as non-coding RNA transcripts longer than 200 nucleotides. First regarded as transcriptional noise, lncRNAs are now known to exhibit diverse functions through a wide array of mechanisms (2,3). In addition, deregulation of lncRNAs is associated with diseases including cancer (4).

The fundamental question of how many lncRNAs are embedded in the human genome has proven to be difficult to answer. While some studies report large transcriptomes containing over 90 000 lncRNAs (5), more conservative resources such as GENCODE annotate only 16 000 lncRNAs (6). The challenges in the annotation of lncRNAs have led to the creation of several specialised lncRNAs databases. Notable examples are LncRNAWiki, a wiki-based resource that combines computational and manual curation (7,8) and NONCODE, a lncRNA annotation database covering 17 species of which human and mouse have the highest number of annotations (9). Of note, lncRNA annotation is not limited to human or laboratory animal species. The domestic-animal lncRNA database ALDB for instance, stores pig, chicken and cow lncRNAs (10). And even though lncRNAs are often regarded as evolutionary new, also plant lncRNAs have been discovered and are catalogued in the PLncDB database (11). A recent valuable effort by the European Bioinformatics Institute (EMBL-EBI) aims to unite all non-coding RNA annotation databases into a single compendium RNAcentral (12).

While the advent of massively parallel RNA sequencing technologies drastically accelerated the identification of novel lncRNAs, functional annotation is lagging behind. In addition, the lack of official gene names for many lncRNAs makes it increasingly difficult to keep track of what is currently known of a particular. Several research groups have therefore turned to manual literature curation to annotate lncRNA with functional evidence or aberrant expression in disease contexts. Notable examples of such datasets are Lnc2Cancer (13), LncRNADisease (14), the recently published pan-cancer lncRNA co-expression atlas LncMAP (15) and the Mammal ncRNA Disease Repository (MNDR) that stores 3213 mammalian lncRNAs associated with diseases (16). Despite these clear advances in lncRNA annotation, current resources are unfortunately still incomplete and plagued with inaccurate transcript and gene models, with import consequences for the lncRNA research field (17). In addition, the coding potential of numerous genes is still debated, and as such the fundamental differentiation between coding and non-coding RNA remains troublesome (18).

In 2012, we released LNCipedia, a database to collect human lncRNA sequences and annotation (19). Central to LNCipedia is the merging of redundant transcripts across the different data sources and grouping of the transcript into genes resulting in a highly consistent database. Through regular updates, LNCipedia offers a complete set of human lncRNAs without compromising the quality of the annotations. An example of this is the high-confidence gene set introduced in LNCipedia 3 (20) as a subset of the database with lncRNAs that lack coding potential by any metric. Here, we describe the development and novel features of LNCipedia 5, the latest update of the database. Following the release of valuable resources such as FANTOM CAT (21), we expanded our database with new lncRNAs. In addition to several small improvements, we introduced an improved filtering pipeline and support for official HGNC gene names. Importantly, an extensive manual literature curation effort resulted in the annotation of 2 482 lncRNA publications, providing insights into functions of 1555 human lncRNAs.

## NEW FEATURES IN LNCIPEDIA 5

### LNCipedia 5 content and filtering

New lncRNA annotation in LNCipedia 5 (Table 1) originates from Ensembl (22), RefSeq (23) and FANTOM CAT (21). In the last few years, the Ensembl lncRNA annotation increased substantially, mainly through the efforts of the GENCODE consortium (24,25). We therefore updated the Ensembl lncRNA annotation to version 92, the latest release at the time of development. Similarly, RefSeq annotation was updated to the NCBI Annotation Release 106. FANTOM CAT (21) lncRNAs constitute the third addition to LNCipedia. This interesting lncRNA resource is based on cap analysis of gene expression (CAGE) data, providing annotation with more accurate 5′ end positions. As a result, almost no overlap between this and other resources can be seen on the transcript level, while 45% of the genes is shared with at least one other source in the database (Table 1, Supplementary Figure S1).

**Table 1.** The different sources of lncRNA transcripts used in LNCipedia 5. The number of unique transcripts and genes varies substantially between the different sources

Source	Full dataset	High-confidence set	Unique transcripts	Unique genes
Broad Institute	14 043	13 277	1627 (12%)	21 (0.3%)
Ensembl release 92 - April 2018	25 075	22 551	8631 (34%)	1334 (10.2%)
FANTOM CAT (stringent)	24 756	22 067	24 750 (100%)	2776 (42%)
NONCODE v4	77 529	62 918	46 124 (59%)	24 901 (55.1%)
Refseq - NCBI Annotation Release 106	5188	4319	2718 (52%)	97 (2.5%)
Sun and Gadad *et al.*, 2015	2124	1672	2124 (100%)	546 (34.3%)
Nielsen *et al.*, 2014	7119	6775	7074 (99%)	6119 (86.7%)
Hangauer *et al.*, 2013	5296	5232	357 (7%)	18 (0.4%)
Total number of unique transcripts	127 802	107 039		
Total number of unique genes	56 946	49 372		

In LNCipedia 5, we also introduced a new filtering pipeline to remove transcripts originating from protein coding genes. Several resources annotate transcripts with truncated or partial ORFs that overlap coding ORFs, often referred to as processed transcripts, as long non-coding RNA. While it is possible that such transcripts are functional, many likely originate from problems in the RNA sequencing data analysis pipeline. We therefore opted to follow a strict definition where transcripts that have exons overlapping with coding ORFs in sense are not regarded as lncRNAs. In this way, 9203 genes were removed, while 455 new genes were added compared to LNCipedia 4 (Supplementary Figure S2). This brings the total number of lncRNA genes in the database to 56 946 (127 802 transcripts), constituting a 13% decrease in the number of genes compared to LNCipedia 4 (65 694 genes). The high-confidence set, a subset of transcripts that lack coding potential by any metric (20), presently contains 49 372 genes (107 039 transcripts), an increase of 5% compared to the 47 213 genes in LNCipedia 4. This was not unexpected as transcripts overlapping annotated protein coding sequence often show increased coding potential and were as such never part of the high-confidence set.

### Improved gene naming includes official gene symbols

In the first version of LNCipedia (19), only very few lncRNAs were annotated with an official gene symbol. Therefore, we introduced at that time a universal scheme that names a lncRNA after the nearest protein coding gene on the same strand. As the number of lncRNAs with a gene symbol provided by the HUGO Gene Nomenclature Committee (HGNC) has grown substantially (26), LNCipedia 5 now uses a hybrid solution for gene names. If an official gene symbol is available, it will be used as the primary identifier in LNCipedia; otherwise, the universal naming scheme will be used. HGNC gene symbols, full names and identifiers are automatically retrieved from the HGNC website and matched to transcripts in the database. Currently, 23% of the transcripts and 6% of the genes in LNCipedia are annotated with an official gene symbol.

### Literature annotation provides insights into functions

**Figure 1.**
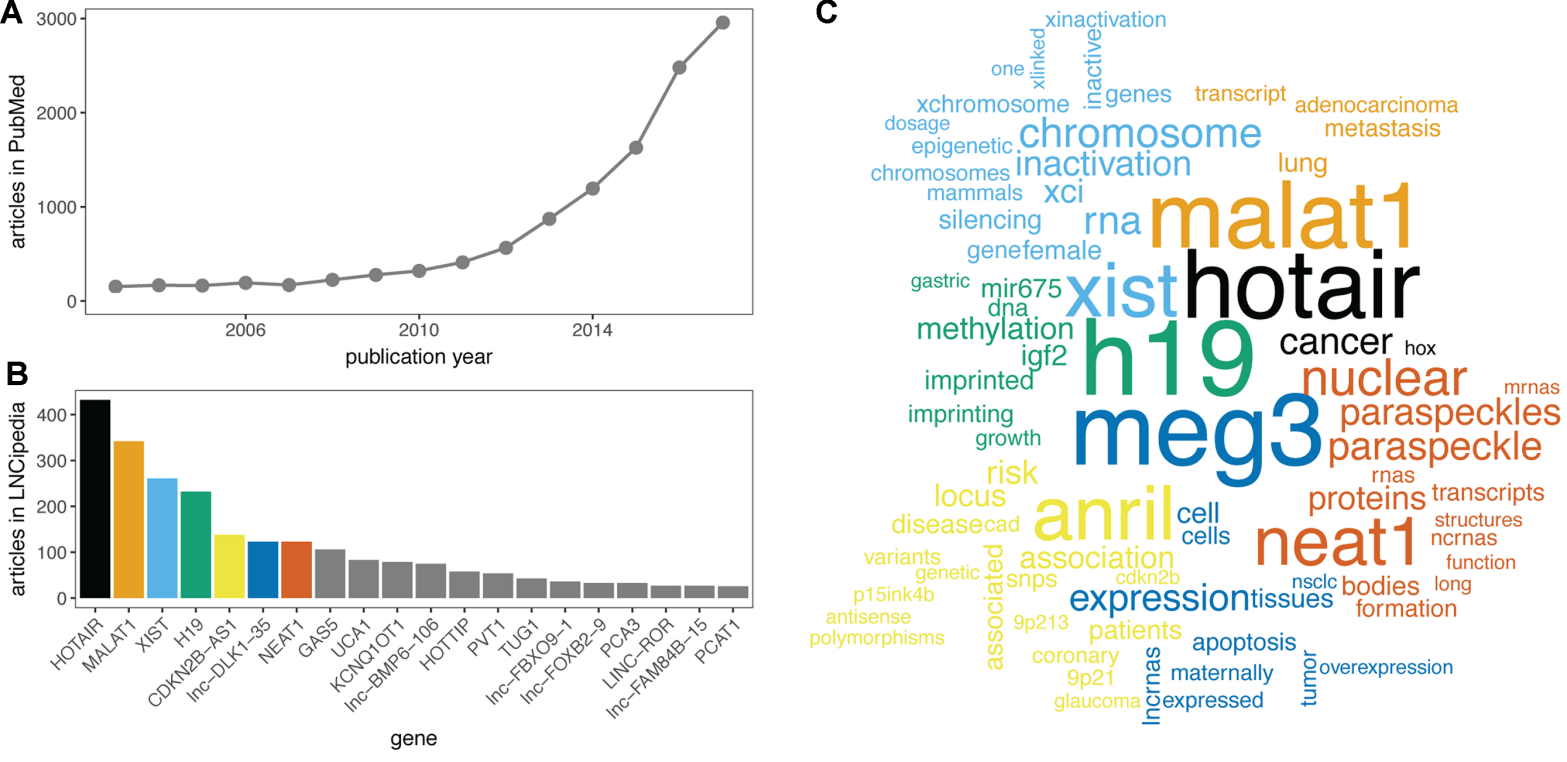
(**A**) The number of publications on lncRNAs has grown rapidly over the past years. Shown here are the number of entries in PubMed with keyword ‘long non-coding RNA’. (**B**) LncRNA functional annotation is heavily skewed towards a small number of lncRNAs. (**C**) Comparison word cloud of the paper abstracts associated with the seven lncRNAs with the most associated papers. Functions and disease associations are immediately clear from this analysis.

Compared to protein coding genes, lncRNAs are poorly understood as the greater majority lacks functional annotation. Nevertheless, the number of PubMed articles with the keyword ‘long non-coding RNA’ has grown enormously of the past years (Figure 1A). Although the available literature is heavily skewed towards a few well-studied lncRNAs (Figure 1B), a few thousand lncRNAs have now been studied functionally. Unfortunately, the lack of official names or stable identifiers, such as Ensembl or RefSeq identifiers, for the majority of lncRNAs had led authors to use their own in-house identifiers. This poses an important challenge for annotators and complicates the functional annotation of lncRNAs. Several annotation groups have therefore used manual curation of lncRNA articles to collect useful resources of functional annotation (13–16). For LNCipedia, we combined manual and programmatical curation of thousands of lncRNA papers in PubMed and linked the papers to entries in our database (supplemental methods). In this way, we were able to associate 2482 PubMed articles with lncRNAs in LNCipedia raising the number of lncRNA genes with at least one published article to 1555. The articles associated with a specific lncRNA are displayed on the transcript and gene pages. In addition, the titles and abstracts of these articles can be searched by keyword.

### Numerous small updates to improve usability

To accommodate users that visit the website on devices with small screens such as smartphones, a new and responsive design was implemented using Bootstrap (https://getbootstrap.com/).

In addition to the GRCh37/hg19 reference genome, LNCipedia now supports GRCh38/hg38 as well. All genomic positions are automatically remapped to both reference genomes (supplemental methods), ensuring the annotations are available with both. The user can choose the desired reference genome and all coordinates on the websites are automatically displayed with respect to that reference genome. In addition, database exports are also available for both reference genome coordinates.

LncRNAs are frequently subclassified based on their relative genomic orientation to protein coding genes (27). LNCipedia 5 uses sequence ontology (SO) terms (28) to annotate lncRNAs according to these subclasses (Table 2).

**Table 2.** Sequence ontology terms used to annotate lncRNA subclasses

Classification	Gene level SO term	Transcript level SO term
intergenic	lincRNA_gene (SO:0001641)	lincRNA (SO:0001463)
antisense	antisense_lncRNA_gene (SO:0002182)	antisense_lncRNA (SO:0001904)
intronic	sense_intronic_ncRNA_gene (SO:0002184)	sense_intronic_ncRNA (SO:0002131)
sense-overlapping	sense_overlap_ncRNA_gene (SO:0002183)	sense_overlap_ncRNA (SO:0002132)
bidirectional	bidirectional_promoter_lncRNA (SO:0002185)	NA

## CONCLUSION AND FUTURE PERSPECTIVES

LNCipedia 5 marks the fifth release of the most comprehensive human lncRNA database, first presented in 2012. These five releases average to almost one mayor release per year, with several small updates in between. While the improved filtering resulted in a smaller set of lncRNAs, we are confident this set will be more useful for researchers that use LNCipedia to annotate their experimental data or design platforms or products based on LNCipedia lncRNAs.

Of note, while previous releases were always accompanied by large increases in the number of lncRNA gene loci, it appears that this number has now more or less stabilised around 55 000. Likewise, lncRNA research in the last years has shifted gears to more accurate annotation (21,25) and providing insights into lncRNA functions. As such, 1555 lncRNA genes in LNCipedia are currently annotated with functional information. While this annotation is far from exhaustive, we hope to improve it drastically over the next years to be able to construct a reference set of well annotated and functionally described lncRNAs.

## DATA AVAILABILITY

LNCipedia 5 is freely available on https://lncipedia.org. The website offers a download section with various exports in BED, GFF, GTF and FASTA format. In addition, a representational state transfer (REST) application programming interface (API) is available to allow programmers to interface with our website programmatically. The API returns documents in the JSON format and can be used in any programming language. In addition, easy integration with the Integrative Genomics Viewer (IGV) (29) is provided, allowing researchers to directly overlay (RNA) sequencing data with LNCipedia annotation. Finally, LNCipedia is also available as a UCSC genome browser track hub (30).

## Supplementary Material

Supplementary Data
