# Supplementary material for: LNCipedia 5: towards a reference set of human long non-coding RNAs
Source: Nucleic Acids Res. 2018 Oct 29;47(Database issue):D135–9. doi: 10.1093/nar/gky1031 (PMC6323963; doi:10.1093/nar/gky1031)
Supplement: Supplementary Data [file gky1031_supplemental_files.docx]

**Supplemental figures**


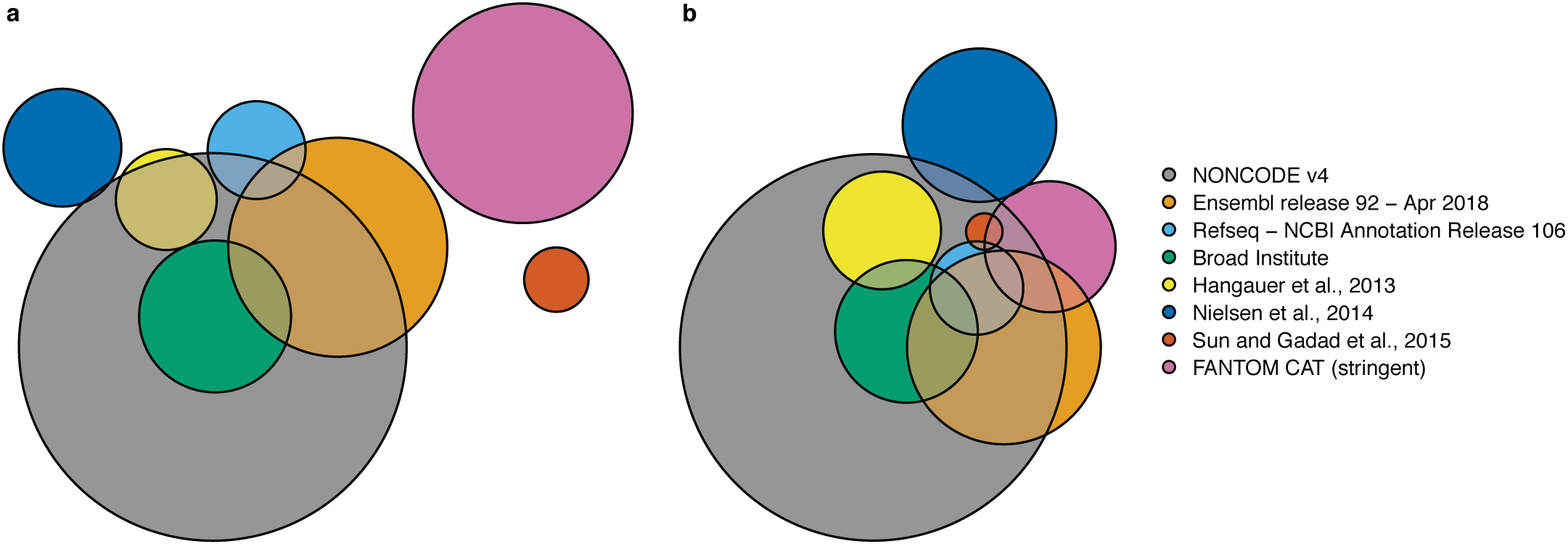


Figure S1. Transcript level overlap differs from gene level overlap for the different sources used for LNCipedia 5. (a) Transcript level overlap between the different sources used for LNCipedia 5. (b) Gene level overlap between the different sources used for LNCipedia 5. While the FANTOM CAT resource has virtually no (< 1 %) transcripts in common with other resources, the gene level overlap is much higher (45 %). Analysis of content overlap demonstrates that different RNA sequencing workflows and data processing pipelines have a great impact on lncRNA transcript annotation.


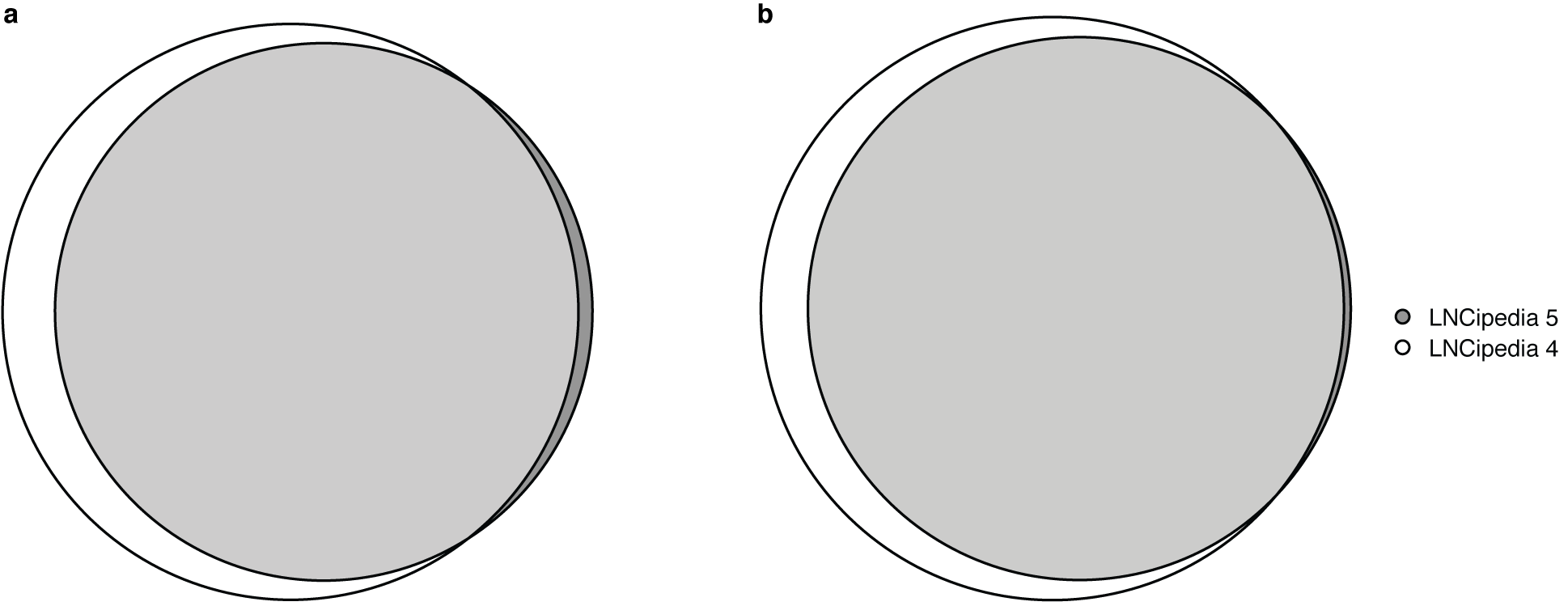


Figure S2. Transcript and gene level overlap between LNCipedia version 4 and 5 shows that relatively more lncRNAs were removed, as a result of the more stringent criteria, than added. (a) Transcript level overlap between LNCipedia 4 and 5. In total, 21 687 transcripts were removed, while only 2 747 were added. (b) Gene level overlap between LNCipedia 4 and 5. In total, 9 203 genes were removed while only 455 new genes were added.

**Supplemental methods**

**Transcript merging and filtering**

First, transcripts from different sources are added to the database using custom import scripts. Two transcripts are merged if all their exon positions are identical, as such creating a nonredundant transcript collection. After each import, the chromosomal positions of the newly added exons are converted to either hg19 or hg38 using the liftOver tool and corresponding chain files provided on the UCSC genome browser website (<https://genome.ucsc.edu/cgi-bin/hgLiftOver>). Only conversions with perfect remapping of all bases are considered. Next, the metadata (conservation, coding potential, HGNC gene symbols, …) for each transcript is either obtained or generated.

In the filtering step, the following transcript are removed from the database:

- Transcripts that do not map to the hg38 reference genome
- Transcripts that are not localized on the reference chromosomes
- Transcripts with less than 200 nucleotides
- Transcripts with exons that overlap coding sequence as annotated in the Ensembl 92 database

Following the filtering, all transcripts are clustered into genes based on exon overlap. These genes are then given a LNCipedia name. Genes containing transcript with official gene symbols are named accordingly. If no official gene symbol is available, a universal scheme that names lncRNAs after the nearest protein coding gene on the same strand is used. The naming schema has been described previously. If the transcripts already have an identifier from a previous version of LNCipedia, those identifiers will be used.

In the final step, transcripts and genes are subclassified based on their relative genomic orientation to protein coding genes (Table 2 main text).

**Literature annotation**

The literature currently in the LNCipedia databases has been obtained from several sources. Firstly, in 2016, a systematic manual curation effort was launched for articles published between 2011 and 2015. The articles where curated by biocuration company E-merge tech (Chennai, India). Briefly: articles with search term “long noncoding RNA” published between 2011 and 2015 were obtained from PubMed (4654 articles). Only articles on human lncRNAs with full text availability were selected for curation. If the article refers to the lncRNA(s) using an official gene symbol or systematic name (such as Ensembl or RefSeq identifiers), those were used to identify the corresponding LNCipedia entries. If no such identifiers are available or the identifier is not found in LNCipedia, chromosomal position, lncRNA sequence or primer sequences were used to find the corresponding LNCipedia entries. In total, 1464 (out of the 4654 in the PubMed search) were added to the database as part of this curation effort. In addition to the systematic curation effort, lncRNA researchers are encouraged to submit articles through the LNCipedia website. Several researchers have used this feature and after evaluation by one of the LNCipedia administrators, the articles were added to the database. A third source of articles in the database are those submitted by members of the LNCipedia development team. These comprise mainly notable lncRNA papers that are submitted on an irregular basis. A final source of lncRNA papers is the HGNC (HUGO gene nomenclature committee) website (<https://www.genenames.org/>). The database is queried as part of the LNCipedia annotation pipeline and relevant articles are automatically retrieved and submitted to the database.
